# Rhamnolipids as a Tool for Eradication of *Trichosporon cutaneum* Biofilm

**DOI:** 10.3390/biom11111727

**Published:** 2021-11-19

**Authors:** Olga Maťátková, Irena Kolouchová, Kristýna Lokočová, Jana Michailidu, Petr Jaroš, Markéta Kulišová, Tomáš Řezanka, Jan Masák

**Affiliations:** 1 Department of Biotechnology, University of Chemistry and Technology, Technická 5, 166 28 Prague, Czech Republic; olga.matatkova@vscht.cz (O.M.); irena.kolouchova@vscht.cz (I.K.); kristyna.lokocova@vscht.cz (K.L.); jana.michailidu@vscht.cz (J.M.); jan.masak@vscht.cz (J.M.); 2 Department of Biochemistry and Microbiology, University of Chemistry and Technology, Technická 5, 166 28 Prague, Czech Republic; petr.jaros@vscht.cz; 3 Institute of Microbiology, Academy of Sciences of the Czech Republic, Vídeňská 1083, 142 20 Prague, Czech Republic; rezanka@biomed.cas.cz

**Keywords:** rhamnolipids, biofilm, *Trichosporon cutaneum*, flow-chamber, eradication

## Abstract

Microbial biofilms formed by pathogenic and antibiotic-resistant microorganisms represent a serious threat for public health in medicine and many industrial branches. Biofilms are involved in many persistent and chronic infections, the biofouling of water and food contamination. Therefore, current research is involved in the development of new treatment strategies. Biofilm is a complex system, and thus all aspects of the measurement and monitoring of its growth and eradication in various conditions, including static and dynamic flow, are issues of great importance. The antibiofilm character of rhamnolipid mixtures produced by four *Pseudomonas aeruginosa* strains was studied under different conditions. For this purpose, the biofilm of opportunistic pathogen *Trichosporon cutaneum* was used and treated under static conditions (microscope glass coverslip in a Petri dish) and under dynamic conditions (a single-channel flow cell). The results show that the biological activity of rhamnolipids depends both on their properties and on the conditions of the biofilm formation. Therefore, this aspect must be taken into account when planning the experimental or application design.

## 1. Introduction

Rhamnolipids are a class of glycolipids, and their molecule is formed by one or two (l)-rhamnose molecules, with a glycosidic linkage to the hydrophobic group made up of one or two β-hydroxy fatty acids. These amphiphilic compounds belong to biosurfactants, produced mainly by microorganisms. The best-studied producer of rhamnolipids is the Gram-negative bacteria *Pseudomonas aeruginosa* [1,2]. The research on rhamnolipids indicates that there are more reasons that microorganisms synthesize these compounds. One of the main functions is to improve hydrophobic substrate uptake. Rhamnolipids serve as mediators in the biodegradation of such substrates both indirectly, by modifying the surface of microbial cells, and directly, by emulsifying hydrophobic substrates [3]. In addition, studies have confirmed that rhamnolipids also play an important role during biofilm formation, from the modification of surface properties to the first steps of cell adhesion up to first microcolonies appear [1]. The function of rhamnolipids is also connected with the formation of transport channels in biofilm and cell release. Their antimicrobial nature helps the producer to compete for the colonization of the environment. The mechanism of action is believed to be based on the solubilization of cell membranes [1,3]. Therefore, rhamnolipids are intensively studied as potential antimicrobial and antibiofilm substances to be used for the treatment of surfaces against drug-resistant pathogens, the development of new pharmaceuticals [4] or the modulation of hydrophobic substrates intake [5].

Biofilm is defined as a highly structured community of microorganisms established in a three-dimensional structure that is irreversibly attached to a surface, an interface or each other. Biofilm cells are enclosed in a matrix of extracellular polymeric substances and exhibit altered phenotypes in comparison with planktonic cells [6]. Biofilms in nature have unique architectural features, including interstitial voids between macro- and microcolonies. The voids allow the diffusion of nutrients, gasses, signal molecules and other substances. The main advantages of biofilm formation include protection from the environment and resistance to physical and chemical stress and thus better resistance and adaptability to various conditions [7]. Therefore, biofilm formation by microbial strains, which are resistant to treatment by antibiotic or antimicrobial substances, represents serious complications in many fields, particularly in the medicine and food industries [8].

There are several experimental platforms suitable for the study of biofilms. Each method has advantages and limitations that must be considered according to the purpose of the study. Experiments performed in static systems (typically microtiter plates or Petri dishes), which employ static cultivation followed by a rinsing step and evaluation of the attached biomass, is the most commonly used method [9]. However, these static systems have several problems, which must be taken into account when the method is used, e.g., definition of magnitude of the rinsing forces applied, definition of loosely adhering cells, and percentage estimation of the total adhering cells that were removed by rinsing [10]. In addition, microtiter experiments are in a batch formation, which results in nutrient exhaustion, unless the media are not replaced. Therefore, this organization is rather suitable for screening experiments [9].

To overcome the above disadvantages, several continuous systems were designed, e.g., rotating disk reactor or modified Robbins device [9]. For the study of cell adhesion under specific hydrodynamic conditions, a flow chamber system was developed. In these devices, the biofilm is cultivated in a closed reactor (flow cell chamber), usually with a window for real-time microscopic analysis. Other advantages of this system are rapid mass transfer, a reduction in mixing time and processing time, the minimization of expensive substrates and a minimal sample volume [11]. Continuous laminar flow also ensures that all planktonic-growing and detached cells are taken away with the medium, and therefore, they do not interfere with the microscopic analysis.

In the presented work, we compared the antibiofilm effects of four rhamnolipid solutions with differing compositions and properties, produced by different strains of *Pseudomonas aeruginosa*. The rhamnolipid activity of *Trichosporon cutaneum* biofilm was studied. *Trichosporon* spp. are basidiomycetous yeast-like anamorphic microorganisms, which are widely distributed in nature. In humans, *Trichosporon* spp. Are occasionally part of the gastrointestinal and oral cavity microflora and can transiently colonize the respiratory tract and skin. Some representatives belong to opportunistic pathogens and can cause invasive disease in immunosuppressive patients. The origin of infections is usually associated with central venous catheters, vesical catheters and peritoneal catheter-related devices. *Trichosporon* cells have the ability to adhere to the surfaces of these materials, colonize them and form biofilms, resulting in invasive trichosporonosis, which is often resistant to conventional therapy [12]. Biofilm eradication of *T. cutaneum* experiments was performed in static systems and in single-channel flow cells, and the effect of rhamnolipids on adherent cells was determined by microscopy and evaluated by image analysis software NIS Elements.

## 2. Materials and Methods

### 2.1. Microorganisms and Culture Media

Yeast *Trichosporon cutaneum* was obtained from the Culture Collection of Yeast, Institute of Chemistry, Slovak Academy of Sciences, Bratislava, Slovakia. Stock culture was stored at −70 °C in 50% glycerol solution. For biofilm cultivation, mineral medium TCM was used (g L^−1^): KH_2_PO_4_ 1.70; Na_2_HPO_4_·2H_2_O 0.75; (NH_4_)_2_SO_4_ 4.00; MgCl_2_·6H_2_O 0.34; MnCl_2_·4H_2_O 0.02; CaCl_2_·2H_2_O 0.02; FeSO_4_·7H_2_O 0.001; NaMoO_4_·2H_2_O 0.001; phenol 0.50.

Rhamnolipid-producing bacteria *Pseudomonas aeruginosa* DBM 3774, *Pseudomonas aeruginosa* DBM 3775, *Pseudomonas aeruginosa* DBM 3776 and *Pseudomonas aeruginosa* DBM 3777 were obtained from the Collection of Yeasts and Industrial Microorganisms (DBM), Department of Biochemistry and Microbiology, University of Chemistry and Technology, Prague. Stock cultures were stored at −70 °C in 50% glycerol solution. The cultivation for rhamnolipid production was carried out in basic mineral medium (g L^−1^): KH_2_PO_4_ 3.4; K_2_HPO_4_ 4.4; NaNO_3_ 15; KCl 1.1; NaCl 1.1; MgSO_4_ 0.224; FeSO_4_ 2.8 × 10^−4^; yeast extract 0.5; ZnSO_4_·7H_2_O 1.45 × 10^−3^; CuSO_4_·5H_2_O 1.25 × 10^−3^; MnSO_4_·H_2_O 8.4 × 10^−3^; CaCl_2_·4H_2_O 1.2 × 10^−3^.

### 2.2. Rhamnolipids Production and Isolation

The cultivation of *P. aeruginosa* for rhamnolipid production was carried out in 500 mL Erlenmeyer flasks (30 °C, 100 rpm) in 200 mL of basic mineral medium, and after the cultivation, the rhamnolipids were isolated (both procedures derived from [13]). Briefly, the biomass was removed by centrifugation (10,000× *g*, 15 min), and the supernatant was subjected to acidic precipitation (1M HCl, 4 °C, 24 h). The precipitate was centrifuged (10,000× *g*, 30 min) and extracted five times by chloroform: methanol solution (1:1) and analyzed by MS. The total rhamnolipid content was determined by measuring the concentration of rhamnose by the phenol-sulfuric method [14] with rhamnose as the standard, which was verified by HPLC (correlation coefficient 0.87). After determining the rhamnolipid composition by MS, the correlation factor between rhamnose (determined by the phenol-sulfuric method) and total rhamnolipids was estimated.

### 2.3. Mass Spectrometry

Isolated rhamnolipids were analyzed by an LTQ OrbitrapVelos mass spectrometer (Thermo Fisher Scientific, San Jose, CA, USA), as described in [13]. ESI-MS analysis was performed in negative ion mode. MS spectra were obtained in FT mode. MS spectra were acquired with a target mass resolution of R = 30,000 at *m*/*z* 400. The ion spray voltage was set at −2500 V (in the negative ionization mode), and the scan-range of the instrument was set at *m*/*z* 150–2000. Nitrogen was used as a nebulizer gas—set at 18 arbitrary units (sheath gas) and 7 units (auxiliary gas). For the CID method, helium was used as a collision gas, and normalization energy of 35% was used for the fragmentation of the parent ions. The tandem MS productions were detected by the high-resolution FT mode. Flow Injection Analysis (FIA) was used for sample introduction into the heated ESI (H-ESI) ion source (250 °C). Acetonitrile–water (50:50, *v*/*v*) was used at a flow rate of 150 µL min^−1^. 

### 2.4. Determination of Critical Micellar Concentration

The critical micelle concentration (CMC) was determined by the measurement of the contact angle (CAM method) [15]. Tested rhamnolipids were diluted in distilled water in the centration range 1–200 mg L^−1^. A 5 µL drop of rhamnolipids solution was placed on a polystyrene plate (constant temperature 25 °C), and a picture of the drop was taken. Each measurement was repeated 10 times. The contact angle was determined by OneAttension software (version 1.8, 2017) in connection with CAM 2008 contact angle goniometer (KSV Instruments, Espoo, Finland).

### 2.5. Biofilm Treatment under Static Conditions

The screening experiments were performed under static conditions in a polystyrene Petri dish (diameter 90 mm) with a microscope glass coverslip (24 × 60 mm) for the monitoring of cell adhesion. Each dish contained 30 mL of culture media, which was inoculated by *T. cutaneum* (OD_400_ = 0.20 ± 0.02). The cultivation was performed at 30 °C and 50 rpm for 24 h when the biofilm surface area reached about 50%. Afterwards, the surfactants were added, and their effect was examined after 2 and 16 h of exposition. The concentration range (1, 5, 10, 100, 250, 500 and 1000 mg L^−1^) including the critical micellar concentration (CMC) was tested to find conditions for analysis under dynamic conditions. For biofilm analysis, the glass slides were carefully removed from the medium and rinsed by a sterile saline solution to remove the loosely attached cells. The effect of surfactants on cell adhesion was determined as described below. For comparison, synthetic surfactants anionic surfactant sodium dodecyl sulphate (SDS, Art. No. 4360.1, Carl Roth, Karlsruhe, Germany) and nonionogenic Tween 80 (Art. No. 9139.1 Carl Roth, Karlsruhe, Germany) were used in addition to the four rhamnolipids. All experiments were performed in triplicate; error bars represent standard deviation.

### 2.6. Biofilm Treatment under Dynamic Conditions

Under the dynamic condition, the biofilm was grown in a single-channel flow cell (Item No. FC 81-PC, BioSurface Technologies, Bozeman, MT, USA) that uses a standard microscope coverslip and microscope glass slide as a viewing window. The corpus of the flow cell is made from an inert material (polycarbonate and black anodized aluminum), which allows repeated use and sterilization. The inner dimensions of flow chamber are 50 × 13 × 2.35 mm. A cell suspension was circulated from a 250 mL stirred Erlenmeyer flask (100 rpm) to the flow cell and then back to the flask. The flow rate was previously tested (data not shown) to obtain conditions enabling cell adhesion and sufficient medium circulation. The optimal conditions were found at a flow rate of 0.067 mL s^−1^ at which the calculation of the Reynolds number (Re = 8.44) confirms laminar flow. For each experiment, 100 mL of TCM media was inoculated by *T. cutaneum* (OD_400_ = 0.20 ± 0.02). Cell adhesion was allowed for 8 h (30 °C) to reach compact biofilm (100% cell adhesion). Subsequently, studied surfactants were added, and their effect was analyzed after 2 and 16 h of exposition. Error bars in Figure 2 indicate the means and standard deviation of at least three independent experiments.

### 2.7. Biofilm Analysis

The analysis of cells adhesion to the surface of a glass microscopic slide (both for static and flow cell cultivation) was carried out by Microscope Nikon Eclipse E400 (Nikon, Tokyo, Japan) equipped with a Canon 1100D digital camera. Acquired images were evaluated by image analysis software (NIS Elements version 3.1, 2010, Nikon, Tokyo, Japan), as described in [16]. At least 20 images of the central part of the glass slide were taken, and images were evaluated by image analysis with the biofilm covered area as the main parameter.

### 2.8. Contact Angle Measurement

Contact angle measurement (CAM method) [15] was used for the determination of *T. cutaneum* envelope hydrophobicity with or without an adsorbed surfactant layer, as well as for the determination of microscope slide glass hydrophobicity. The treatment was performed with surfactants at their critical micelle concentration. Cells were cultivated in 500 mL Erlenmeyer flasks in TCM medium (30 °C, 100 rpm). After 48 h of cultivation (exponential phase), cells were harvested by centrifugation (9000× *g*, 10 °C, 10 min), washed, resuspended in TCM medium (final OD_400_ = 1.60 ± 0.02) and incubated (30 °C, 100 rpm) with surfactant for 2 h. Suspension was harvested by centrifugation (9000× *g*, 10 °C, 10 min), washed and coated on a clean glass microscopic slide. The contact angle of Millipore water (drop volume 2 ± 0.1 µL) on a microbial surface was measured by a KSV CAM 200 goniometer (KSV Instruments, Espoo, Finland). At least six droplets were placed in the different positions, and results were statistically evaluated using the Dean–Dixon (Q) test (α = 0,05, Q_crit_ = 0.371).

## 3. Results

Rhamnolipid mixtures produced by four *Pseudomonas aeruginosa* strains were studied for their potential impact on the formation of *Trichosporon cutaneum* biofilm. The properties and antimicrobial/antibiofilm character of rhamnolipid mixtures are strongly dependent on their composition. In the presented work, four strains of *P. aeruginosa* were used for rhamnolipid production, and the composition of isolated rhamnolipid mixtures was analyzed. The proportion of rhamnolipid congeners, which is listed in Table 1, significantly varied between the strains. The rhamnolipid mixtures consist mainly of four types of congeners, which are formed by one or two molecules of rhamnose (Rha) and one or two molecules of fatty acids (FA)—these are mono-rhamnolipids, RhaFA and RhaFAFA, and di-rhamnolipids, RhaRhaFA and RhaRhaFAFA. From our results, the level of congeners in rhamnolipid mixture (rh) produced by the strain 3776 was the most balanced with respect to all rhamnolipid types. On the contrary, the mixture rh3774 comprised mainly RhaFAFA (50.2%) and RhaRhaFAFA (47.3%) congeners. Mixtures rh3775 and rh3777 showed similar congener type composition, the most abundant congeners being RhaFAFA (48.6% and 44.2%, respectively) and RhaRhaFA (20.7% and 35.1%, respectively). Congener type RhaFA was the least represented in all studied rhamnolipid mixtures (0.5–17.1%).

In relation to the properties of rhamnolipid mixtures, the amount of unsaturated FA among the rhamnolipid congeners is also very significant. The highest proportion of unsaturated FA was found in rh3777 (21.6%) and rh3774 (18.8%). On the contrary, the lowest representation was in rh3776 (5.4%). The content of unsaturated FA and the composition of congeners play an important role in the critical micelle concentration (CMC) of the rhamnolipid mixture. Briefly, CMC is an important parameter characterizing surface active substances and shows when a surface active substance (e.g., rhamnolipid) can spontaneously form stable micelles [17]. The values of CMC are summarized in Table 2. From rhamnolipid mixtures, the highest CMC was detected in rh3774 (75.5 mg L^−1^). On the contrary, the lowest value of CMC was found in rh3776 (15 mg L^−1^). For a comparison of the rhamnolipid impact, anionic (SDS) and nonionogenic (Tween 80) synthetic surfactants were used. The CMC of Tween 80 (13 mg L^−1^) is similar to the CMC of rh3776. The CMC of SDS is very high (above 1000 mg L^−1^).

**Figure 1:**
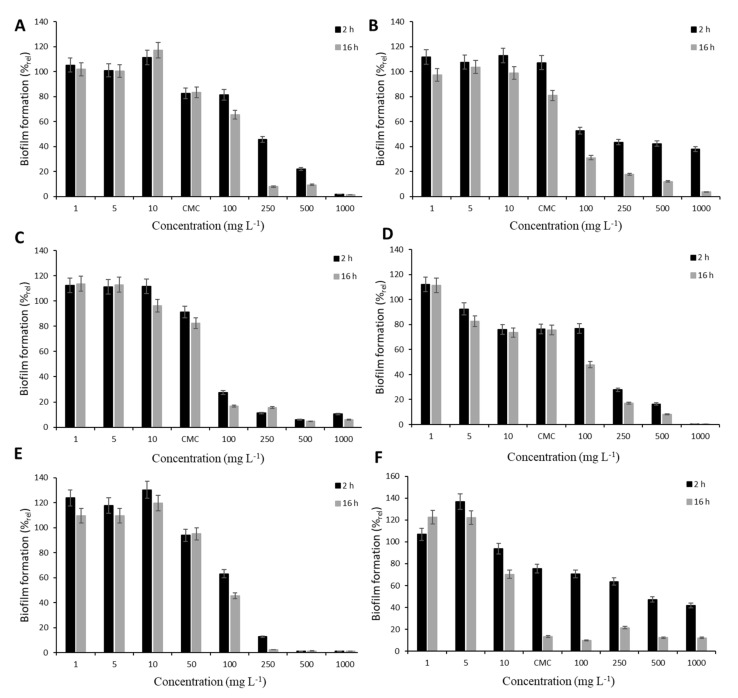
The effect of different surfactants on 24 h mature biofilm under static conditions. The relative percentage expresses the change in biofilm coverage after 2 and 16 h of treatment by rh3774 (**A**); rh3775 (**B**); rh3776 (**C**); rh3777 (**D**); SDS (**E**) *; Tween 80 (**F**). Error bars indicate standard deviation. * The CMC of SDS was not studied due to its high value (>1000 mg L^−1^).

The antibiofilm character of studied surfactants was investigated on the biofilm formation of the opportunistic pathogen *T. cutaneum* biofilm. For screening experiments, static conditions were used, and the biofilm was treated using a concentration range of tested surfactants including CMC (except for SDS, due to its high CMC; see Table 2) for 2 and 16 h. The influence of treatment conditions on mature biofilm is documented in Figure 1. In relation to the diverse composition of rhamnolipid mixtures, different effects on biofilm formation were observed. At concentrations below CMC, no significant decrease in colonization was observed, either after 2 or 16 h. The only exception was detected after biofilm exposition to rh3777. Decreases of almost 10% and 20% after 2 and 16 h, respectively, in colonization were observed after treatment by 5 mg L^−1^. In all cases, the application of rhamnolipids at their CMC led to the restriction of biofilm formation. The highest decrease was elicited by rh3777 (almost 26%). Concentrations higher than CMC subsequently resulted in significant inhibition of biofilm formation. Rhamnolipid rh3776 affected *T. cutaneum* colonization the most; 100 mg L^−1^ elicited decreases of 73% and 83% after 2 and 16 h, respectively. Other rhamnolipids decreased colonization by only 20–50%. All tested rhamnolipids showed similar action to SDS. On the contrary, the behavior of Tween 80 was different. The efficacy after 2 h of exposure to CMC and higher concentrations was significantly lower than after 16 h treatment. Nevertheless, Tween 80 also proved the ability to restrict biofilm formation.

**Figure 2:**
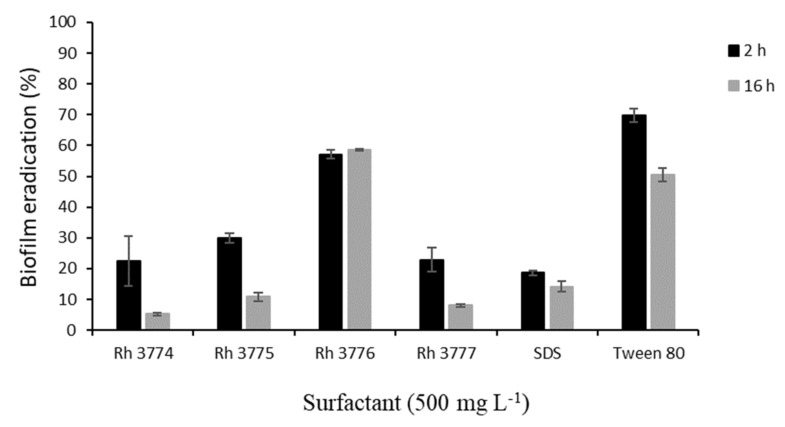
Percentage of biofilm remaining after submitting the biofilm (8 h mature biofilm, 100% surface coverage) to studied surfactants (500 mg L^−1^) for 2 and 16 h. Error bars indicate the means ± SD of several independent experiments.

**Figure 3:**
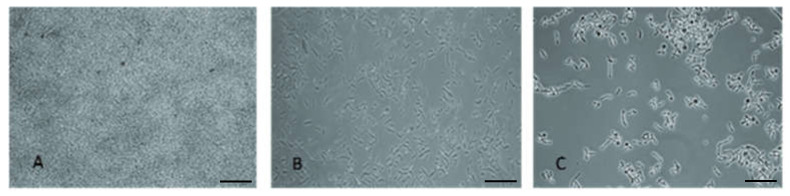
Light microscopy images of *T. cutaneum* dynamic biofilm treatment in the flow cell experiment: (**A**) control, (**B**) after 2 h of rhamnolipid rh3777 treatment, (**C**) after 16 h of rhamnolipid rh3777 treatment. Scale bar 100 µm.

From obtained results, concentration 500 mg L^−1^ caused the inhibition of biofilm formation at least by 80% (16 h). Therefore, this concentration was chosen for further experiments in dynamic conditions. Mature biofilm of *T. cutaneum* was treated by studied surfactants in a single-channel flow cell for 2 and 16 h. The subsequent biofilm eradication is depicted in Figure 2. In all cases, a reduction in biofilm was observed. The highest effect was observed after 16 h application of rh3774 and rh3777, where biofilm reductions of almost 95% and 92%, respectively, were found. On the contrary, rh3776 showed the least impact on mature biofilm (decrease of only about 43%). Representative light microscopy images of the effect of rh3777 on biofilm reduction is depicted in Figure 3. In addition, the duration of the experiment had no effect on biofilm reduction using SDS. However, SDS decreased colonization by almost 86% (16 h). Conversely, Tween 80 also showed low effects like rh3776. The biofilm was eradicated by 30% and 51% after 2 and 16 h, respectively.

Rhamnolipids significantly influence the hydrophobicity of exposed surfaces. Their presence changes not only the hydrophobicity of the cell surface but also of the colonized surface, both of which significantly influence the potential for adhesion. The values of contact angles characterizing the changes in hydrophobicity are summarized in Table 3. The measurement showed that the treatment of cells or surfaces (glass slides) by studied rhamnolipid mixtures at their CMC resulted in a decrease in hydrophobicity. Nevertheless, the extent of the change depended on the used rhamnolipid. Cells of *T. cutaneum* (contact angle 66°) and glass slides (contact angle 63°) evinced similar characteristics of hydrophobicity. The treatment of cells by rhamnolipids rh3776 and rh3777 caused the same change (contact angle 39°, i.e., lower hydrophobicity). On the contrary, their effect on glass slides was significantly different. The highest decrease in *T. cutaneum* hydrophobicity was found after treatment by rh3775 (contact angle 28°), and was the smallest after treatment with rh3774 (contact angle 48°). The influence of synthetic surfactants on the cell surface was minimal.

Results obtained after glass slide treatment showed a very significant influence of all surfactants, with the exception of Tween 80 (contact angle 49°). Interestingly, after treatment by rhamnolipid mixtures rh3774, rh3775, rh3777 and SDS, very low values of contact angles were detected (5–8°). Meanwhile, the effect of rhamnolipid mixture rh3776 was not very distinct (contact angle 24°).

## 4. Discussion

Rhamnolipids have many advantageous properties, which are used in solving problems of environmental pollution; in the food, cosmetics and agricultural industries; and finally, in pharmacy or medicine. In these industries, emulsifying, solubilizing or wetting properties are mainly used, as well as metal sequestration [18] and possibly antimicrobial activity in medicine [19]. These properties are underlined by the environmental friendliness of rhamnolipids. Rhamnolipids are biodegradable substances and, therefore, when applied, we do not encounter toxicity and accumulation in the environment, and thus have huge potential for use in bioremediation [20].

The antimicrobial and antibiofilm activity of rhamnolipids has been reported in many publications [21,22,23,24]. The rhamnolipid antibacterial activity against a wide variety of microorganisms has been reported in many studies [19,24,25]. The mechanism of action of rhamnolipids is complex and mainly involves interactions with cell surface structures such as lipopolysaccharides, phospholipids and proteins [26,27,28]. Rhamnolipids interact with lipopolysaccharides [27], the phospholipid membrane and protein structures [26]. The action of rhamnolipids results in a change in the character of the cell surface, for example, a change in hydrophobicity [27] and / or the surface charge of the cell [29].

Additionally, the effectiveness of action is dependent on the composition of the rhamnolipid mixture, as well as on the type of exposed microorganism [27]. Studied rhamnolipid mixtures produced by four different strains of *P. aeruginosa* showed significantly different representations of congeners, comprising RhaFA, RhaFAFA, RhaRhaFA and RhaRhaFAFA. The presence of unsaturated FA was also determined for the properties of rhamnolipid mixtures. In studied mixtures, the abundance of unsaturated FA varied from 5.4% to 21.6%. Rooney et al. [30] reported similar differences between rhamnolipid mixtures produced by several *P. aeruginosa* strains. The content of unsaturated FA in these mixtures varied between 0% to 12.7%.

The composition of rhamnolipids is crucial for the value of the critical micelle concentration (CMC). Among other factors, CMC increases with the amount of unsaturated FA [31]. On the other hand, the higher content of congeners containing only one molecule of rhamnose (RhaFA and RhaFAFA) results in a decrease in CMC [32]. This correlates with our results, in which the lowest content of unsaturated FA (5.4%) was found in rh3776, as well the lowest value of CMC (15 mg L^−1^). On the contrary, the highest content of unsaturated FA was found in rh3777 (21.6%), but the value of CMC (55.4 mg L^−1^) was lower than in the rh3774 mixture (unsaturated FA 18.8%, CMC 75.5 mg L^−1^). This was probably influenced by the higher abundance of mono-rhamnolipid congeners in rh3777 in comparison with rh3774.

From the screening experiments under static conditions, it is obvious that the composition of rhamnolipids influenced the response of *T. cutaneum* biofilm to the treatment. Interactions between cell surface structures and rhamnolipids are probably a crucial step for the mechanism of action. In addition, the production or presence of rhamnolipids has an important role in the development of biofilm, including the maintenance of open channels and void spaces, as well as the facilitation of cell detachment from the biofilm structure [33]. It was found that a concentration higher than CMC must be used to produce a significant decrease in the colonized area. When the biofilm was exposed to the highest concentration of surfactants (1000 mg L^−1^), removal of more than 90% was achieved. However, the effectiveness of concentrations between CMC and 1000 mg L^−1^ varied. Obviously, the suitability of rhamnolipid mixtures and the used concentration must be studied before concrete application. For example, rh3777 was proven to have a significant impact already at CMC concentration (55.4 mg L^−1^), and the biofilm was reduced by almost 33%. Moreover, the treatment by 250 mg L^−1^ resulted in a decrease of almost 80%. Conversely, rh3776 showed the same effect after biofilm exposition to 100 mg L^−1^, and from this point of view, rh3776 seemed to be the most effective mixture. However, at the CMC, it had a very low impact (9–18%), which may be attributed to the low CMC value (15 mg L^−1^), showing that the absolute concentration value was a more important factor than the CMC. Singh et al. [34] also showed the dependency of rhamnolipid action on concentration when biofilm of *Candida albicans* was treated by rhamnolipids produced by *P. aeruginosa* in a concentration range of 40–5000 mg L^−1^.

Kim et al. [35] reported that the CMC (240 μg mL^−1^) of used rhamnolipids demonstrated efficacy on *P. aeruginosa* biofilm. The anti-adhesive activity of rhamnolipid produced by *P. aeruginosa* against several bacterial and yeast strains isolated from voice prostheses was evaluated in [36]. The experiments were performed under dynamic conditions in a parallel plate flow chamber. The best results for the reduction in the adhesion rate occurred for *Streptococcus salivarius* GB 24/9 and *Candida tropicalis* GB 9/9 (an average of 66%). The potential of rhamnolipids to prevent biofilm formation was reported by Gomes and Nitschke [37]. The treatment by rhamnolipids (1.0% solution) reduced adhesion to the polystyrene of *Listeria monocytogenes* (by 57.8%) and *Staphylococcus aureus* by (67.8%). Dusane et al. [22] showed that rhamnolipid disrupted the pre-formed biofilm of *Yarrowia lipolytica* in a more effective manner than chemical surfactants (cetyl-trimethyl ammonium bromide and SDS).

The conduction of pilot experiments under static conditions was chosen due to its simplicity, cost efficiency and multiplicity. On the other hand, these conditions also have several limitations, including problems with the separation of attached and loosely attached cells, the definition of the washing process and the quantification of washed-off cells [10]. Concurrently, it must be taken into account that the process of biofilm development is very stochastic; therefore, the independent repetition of biofilm cultivation may vary, even if the cultivation conditions are kept constant [38]. In addition, culture conditions are changed over the duration of the experiment (substrate utilization and cell metabolism). These factors could have a significant effect on biofilm stability and further eradication. Therefore, the antibiofilm activity of rhamnolipids was also investigated under dynamic conditions conducted in a single-channel flow cell. The behavior of rhamnolipids and synthetic surfactants did not correspond with those obtained from static conditions in all cases. Rh3774, rh3775 and rh3777 had the same ability to reduce biofilm colonization, up to 95% (16 h). The same effect on biofilm eradication was found after SDS treatment, almost of 86% (16 h). However, rh3776 and Tween 80 showed a different effect; the colonized area was reduced by only 41% and 59%, respectively. These differences in surfactant effectiveness support the necessity to perform experiments under both static and dynamic conditions and highlight the importance of rhamnolipid mixture composition determination and characterization in relation to their intended application. Performed experiments showed that dynamic conditions had no impact on the biological activity of rhamnolipids with higher CMC as well SDS, in contrast to rh3776 and Tween 80, with very low CMC (15 and 13 mg L^−1^, respectively), which were very effective in biofilm eradication. 

Rhamnolipids with low CMC have hydrophobic characteristics, as their molecules are formed predominantly by mono-rhamnolipid congeners with a low abundance of unsaturated FA [27]. The amphiphilicity of rhamnolipid molecules is crucial for the interactions with the structures of the cell surface or colonized area. Adsorbed rhamnolipids thus change the surface charge, resulting in varied microbial adhesion ability [22]. In addition, interactions with proteins or lipids forming the surface of cells can lead to the alteration of cell permeability [39], resulting in a direct impact on cell viability. The treatment of *T. cutaneum* by rhamnolipids caused a decrease in the hydrophobicity of cells. Chrzanowski et al. [40] reported a decrease in the hydrophobicity of yeast *Candida maltosa* after treatment by rhamnolipids (150 mg L^−1^). The same effect of rhamnolipids on food pathogenic bacteria was found by Gomes and Nitschke [37]. Similarly, the conditioning of a glass microscope slide led to a significant decrease in surface hydrophobicity depending on rhamnolipid CMC, and thus rhamnolipid composition.

Differences between results obtained under static and dynamic conditions suggest that biofilm eradication under static conditions is mostly a function of rhamnolipid properties (composition), whereas under the dynamic condition, the eradication is influenced by rhamnolipid properties and medium flow rate. The flow rate of the medium can detach weakly bound cells, which are able to adhere under static condition.

## 5. Conclusions

Many studies have reported the possibility of using rhamnolipids as antibiofilm agents. However, rhamnolipid composition and properties must be studied in detail with respect to their potential application, with a focus on the target microorganism, surface properties and environmental conditions. Our results indicate that rhamnolipid may be used for *Trichosporon* biofilm disruption. After treatment with rhamnolipids at a concentration of 1000 mg L^−1^, the removal of more than 90% of the colonized area was reached. It was also found that rhamnolipids significantly change the hydrophobicity of the microbial cell surface and the glass carrier. From our results, it is obvious that the effect of rhamnolipids in static conditions could differ significantly in comparison with dynamic conditions. This finding supports the necessity to take into account the character of the application area, and thus an appropriate experiment design is crucial in biosurfactant studies. Confirmation of this conclusion would allow the wider use of rhamnolipids, not only as substances promoting hydrophobic substance bioavailability, but also as compounds directly influencing biofilm formation.

## Figures and Tables

**Table 1:** Total relative content of rhamnolipid congeners in rhamnolipid mixtures (rh) produced by four *Pseudomonas aeruginosa* strains (DBM 3774, DBM 3775, DBM 3776 and DBM 3777); Rha—rhamnose; FAfatty acid.

rh Composition (%)	rh3774	rh3775	rh3776	rh3777
RhaRhaFAFA	50.2	17.8	21.8	13.6
RhaFAFA	47.3	48.6	29.7	44.2
RhaFA	0.5	12.9	17.1	7.1
RhaRhaFA	2.0	20.7	31.4	35.1
unsaturated FA content	18.8	13.5	5.4	21.6

**Table 2:** Critical micelle concentrations (CMC) of tested rhamnolipid mixtures and synthetic surfactants.

Surfactant	CMC (mg L^−1^)
rh3774	75.5
rh3775	24.4
rh3776	15.0
rh3777	55.4
Tween 80	13.0
SDS	>1000

**Table 3:** The effect of surfactants at their critical micelle concentration on hydrophobicity of *T. cutaneum* cells and glass microscope slides determined as values of water contact angles. Control represents cells or slide without treatment.

	Surfactant	Contact Angle (°)
*T. cutaneum*	control	66 ± 3
	rh3774	48 ± 2
	rh3775	28 ± 1
	rh3776	39 ± 3
	rh3777	39 ± 2
	SDS *	52 ± 2
	Tween 80	66 ± 1
Glass microscope slide	control	63 ± 1
	rh3774	5 ± 1
	rh3775	6 ± 2
	rh3776	24 ± 2
	rh3777	6 ± 2
	SDS *	8 ± 2
	Tween 80	49 ± 4

* as CMC of SDS is too high, concentration 50 mg L^−1^ was used.

## Data Availability

Not applicable.
